# Antitumor and Antimetastatic Effect of Small Immunostimulatory RNA against B16 Melanoma in Mice

**DOI:** 10.1371/journal.pone.0150751

**Published:** 2016-03-16

**Authors:** Tatyana O. Kabilova, Aleksandra V. Sen’kova, Valeriy P. Nikolin, Nelly A. Popova, Marina A. Zenkova, Valentin V. Vlassov, Elena L. Chernolovskaya

**Affiliations:** 1 Institute of Chemical Biology and Fundamental Medicine SB RAS, 8, Lavrentiev Avenue, Novosibirsk, Russia, 630090; 2 Institute of Cytology and Genetics SB RAS, 10, Lavrentiev Avenue, Novosibirsk, Russia, 630090; Istituto Superiore di Sanità, ITALY

## Abstract

Small interfering RNAs, depending on their structure, delivery system and sequence, can stimulate innate and adaptive immunity. The aim of this study was to investigate the antitumor and antimetastatic effects of immunostimulatory 19-bp dsRNA with 3’- trinucleotide overhangs (isRNA) on melanoma B16 in C57Bl/6 mice. Recently developed novel cationic liposomes 2X3-DOPE were used for the *in vivo* delivery of isRNA. Administration of isRNA/2X3-DOPE complexes significantly inhibits melanoma tumor growth and metastasis. Histopathological analysis of spleen cross sections showed hyperplasia of the lymphoid white pulp and formation of large germinal centers after isRNA/2X3-DOPE administration, indicating activation of the immune system. The treatment of melanoma-bearing mice with isRNA/2X3-DOPE decreases the destructive changes in the liver parenchyma. Thus, the developed isRNA displays pronounced immunostimulatory, antitumor and antimetastatic properties against melanoma B16 and may be considered a potential agent in the immunotherapy of melanoma.

## Introduction

Malignant melanoma is one of the most aggressive forms of skin cancer and is responsible for 80% of mortality from skin tumors [1]. The standard methods used for melanoma treatment include surgical removal, chemotherapy and radiotherapy; however, the rate of long-term survival of patients with melanoma is not encouraging due to its chemoresistance and rapid metastasis [2]. As melanoma is an immunogenic tumor, strategies to enhance the immune response to the tumor have been developed. Recombinant interferon-α (IFN-α) is the basic therapy approved currently for use either alone or as a part of the combined therapy in the treatment of a range of tumors including malignant melanoma [3–5], but this treatment is associated with significant toxicity and the risk of autoimmune reactions [6].

The innate and adaptive immune response can be activated by exogenic nucleic acids (e.g. bacterial, viral and fungal nucleic acids) via several families of pattern recognition receptors, such as endosomal and cell surface Toll-like receptors 3/7/8/9 (TLRs) [7]. TLR ligands are attractive adjuvant candidates for therapeutic cancer vaccines, since TLR signaling stimulates both humoral and cellular immune responses mediated by dendritic cells (DCs). *In vitro* studies have shown that among TLR agonists, nucleic acids can stimulate antitumor responses more effectively than other TLR ligands (e.g. lipoproteins or lipopolysaccharides) [8]. The other group of cellular sensors of nucleic acids includes cytosolic receptors: retinoic acid inducible gene-I-like helicases (RIG1) and melanoma differentiation-associated gene 5 (MDA5); interferon-induced, double-stranded RNA-activated protein kinase (PKR); 2′-5′-oligoadenylate synthase 1 and 2 (OAS); and less studied NOD-like receptors (NLRP3, NOD2) [9, 10]. Based on published data, at least four signaling pathways, including the RIG-I/MDA5 pathway [11, 12], the TLR3 pathway [13, 14], the TLR7/8 pathway [15, 16] and the PKR pathway [17, 18], may recognize small RNAs complexed with cationic delivery vehicles and induce the production of type I IFNs and proinflammatory cytokines.

The activation of cytoplasmic or endosomal sensors of foreign nucleic acids results in the activation of production of proinflammatory cytokines and interferons, the stimulation of the complement system, coagulation, opsonization, phagocytosis and, eventually, apoptosis of tumor cells. There is evidence that induction of IFNα by TLRs has an immunostimulatory effect through activation of natural killer (NK) cells, macrophages and DCs [19]. In their turn, mature DCs can directly interact with immune effector cells such as cytotoxic T lymphocytes (CTLs) and accelerate the clearance of tumor cells by regulating T-cell response [20]. The antitumor activity of TLR 3/7/8/9 agonists has been demonstrated in several tumor types including melanoma, glioblastoma, renal cell carcinoma, and breast, colon and ovarian cancers [21].

Recently, a series of specific unmodified 19-bp dsRNAs with 3’- trinucleotide overhangs (immunostimulatory RNA–isRNA) that possess pronounced antiproliferative activity with respect to various cancer cells and immunostimulatory activity in immunocompetent cells was designed and the consensus sequence of the RNA (Patent of RF № 2391405) was identified [22, 23]. This isRNA induced IFN-α and to a lesser extent proinflammatory cytokine interleukin-6 (IL-6) production in murine blood serum, efficiently reduced the metastases area in different murine organs and slightly inhibited tumor growth in a hepatocellular carcinoma G-29/CBA/LacSto murine model [24].

Here isRNA was applied for treatment of B16 melanoma in C57Bl/6 mice. isRNA delivered into mice in the complex with novel 2X3-DOPE liposomes efficiently inhibits the primary tumor growth and metastasis. Given the duration of the interferon refractory state we optimize the treatment regimen and significantly increase the efficiency of therapy of melanoma B16.

## Materials and Methods

### isRNA

Oligoribonucleotides (strand 1: 5’-AAAUCUGAAAGCCUGACACUUA-3’ and strand 2: 5’-GUGUCAGGCUUUCAGAUUUUUU-3’) were synthesized on an automatic ASM-800 DNA/RNA synthesizer (Biosset) using ribo-β-cyanoethyl phosphoramidites (Glen Research, USA). After standard deprotection, oligoribonucleotides were purified using denaturing polyacrylamide gel electrophoresis (PAGE) and isolated as sodium salts. Oligoribonucleotides were characterized by MALDI-TOF mass spectra on REFLEX III (Bruker Daltonics, Germany). isRNA duplex was annealed at a 50 μM concentration of each strand in a buffer containing 15 mM HEPES-KOH (pH 7.4), 50 mM potassium acetate and 1 mM magnesium acetate.

### Mice and injection of isRNA

All animal procedures were carried out in strict accordance with the recommendations for proper use and care of laboratory animals (ECC Directive 86/609/EEC). The protocol was approved by the Committee on the Ethics of Animal Experiments of the Administration of the Siberian Branch of the Russian Academy of Sciences. Eight- to 12-week-old female C57Bl/6 mice with an average weight of 16–20 g from the vivarium of the Institute of Cytology and Genetics SB RAS were used.

Novel cationic liposomes 2Х3-DOPE used for the delivery of isRNA to their target cells were prepared by Dr. M. Maslov (Lomonosov Moscow State University of Fine Chemical Technology, Moscow, Russian Federation) as described in [25]. The complexes of cationic liposome and isRNA were formed in a serum-free Opti-MEM medium (Invitrogen, USA) via the mixing of equal volumes of liposome solution (final concentration 150 μM) and isRNA solution (final concentration 3.5 μM), followed by incubation for 20 min at room temperature. The phosphate to nitrogen ratio (P/N) in isRNA/2Х3-DOPE complexes was 1/4. The complexes (10 μg of isRNA per mouse) were injected in 200 μl of OptiMEM peritumorally (here and after p.t.) or intravenously (here and after i.v.) into mice.

### Analysis of IFN-α level in murine blood serum

The mice were injected intravenously with 200 μl of isRNA/2X3-DOPE complexes in sterile OptiMEM medium once or twice and the blood was collected 6 hours after the last injection via head clipping. The time intervals between injections varied from 16 to 96 h. The serum was prepared from the whole blood by coagulation for 30 min at 37°C and subsequent centrifugation, and the levels of IFN-α were measured using sandwich ELISA kits (BD Biosciences, USA) in accordance with the manufacturer’s instructions. The IFN-α levels were measured in duplicate in individual serum samples from three mice per group.

### Tumor models and treatment

Melanoma B16 cells were obtained from the N. N. Blokhin Cancer Research Center, Moscow, RF. Two models of melanoma B16 progression in C57Bl/6 mice were used. The model of primary tumor growth (hereafter “subcutaneous melanoma”) was induced by subcutaneous (hereafter “s.c.”) inoculation of B16 cells (1.5 × 10^5^ cells per mouse) into the withers of each mouse. The metastatic melanoma model was induced by i.v. injection of B16 cells (2 × 10^5^ cells per mouse). Tumor cells were transplanted in 100 μl of saline buffer. After tumor transplantation, the animals were assigned to experimental and control groups (10 mice per group) and were then kept in their compartments until the end of the experiment. The isRNA/2X3-DOPE complexes in sterile OptiMEM (200 μl) were injected into the mice as described above. Control mice received the same volume of 2X3-DOPE solution (Mock) or OptiMEM (Control).

The metastatic melanoma was treated by i.v. administration of isRNA/2X3-DOPE on days 2, 6 and 10 after tumor challenge. On day 15 after tumor transplantation the mice were euthanized, and the lungs, livers, kidneys and spleens were isolated and fixed in 4% neutral-buffered formaldehyde for histological examination and morphometry. The number of macroscopically visible surface metastases in the lungs, livers and kidneys was counted.

Mice with subcutaneous melanoma received i.v. or p.t. injections of isRNA/2X3-DOPE on days 8, 12 and 16 after tumor transplantation. Tumor size was measured with calipers on days 7, 11, 14 and 16. Tumor volume was calculated using the formula π/6×(length × width × height). The tumor-bearing mice were examined on day 17 after tumor implantation using T2-weighted MR imaging. Т2-weighted images were obtained with an ultra high field Bio Spec 117/16 Bruker tomograph, using the multislice, multispin echo (MSME) technique. Tumor size reaching 15 mm in any dimension was selected as the ethical endpoint. On day 18 after the tumor transplantation the mice were euthanized, and the lungs, livers, kidneys and spleens were isolated and fixed in 4% neutral-buffered formaldehyde solution for further analysis.

### Histology and morphometry

The fixed murine kidneys, lungs, livers and spleens were routinely processed and embedded in paraffin. Paraffin sections (5 μm) were stained with hematoxylin and eosin according to standard protocols, microscopically examined and scanned. Images were obtained using an Axiostar plus microscope equipped with an Axiocam MRc5 digital camera (Zeiss, Germany). The percentages of the internal metastases area and the splenic follicles area were determined relative to the total area of sections using Adobe Photoshop Software from five random fields of view (100× magnification).

Stereological quantification of the liver sections was performed by point counting, using a framed counting grid at a magnification of 400×. The counting grid had 100 testing points in the studied area equal to 3.2 × 10^6^ μm^2^. Ten to 15 random fields were studied in each specimen. The volume densities (Vv) of normal liver parenchyma, hepatocytes with degenerative and necrotic changes and the numerical density (Nv) of binuclear hepatocytes reflecting the regeneration capacity of the liver were evaluated as described in [26].

### Statistics

The statistical significance of the differences in IFN-α production was determined using the two-tailed Student’s t-test (data are expressed as mean ± SD). The nonparametric Mann-Whitney U test was used for the analysis of the tumor size or weight and metastases number or total area to compare the mean values between the two groups (data are expressed as mean ± SEM). Differences were considered statistically significant at P<0.05.

## Results

### Optimization of isRNA treatment regimen

We applied cationic liposomes (2X3-DOPE) consisting of the recently created polycationic lipid 2X3, built from two cholesterol residues linked with spermine, and lipid-helper DOPE (1,2-dioleoyl-sn-glycero-3-phosphoethanolamine) for the functional delivery of isRNA *in vivo*. Synthesis and the physicochemical characteristics of the liposomes and their complexes with isRNA were published recently [25]. Here we treated the mice with complexes composed of 10 μg isRNA and liposomes 2X3-DOPE at concentration corresponding to P/N (phosphate to nitrogen) ratio 1/4.

**Fig 1:**
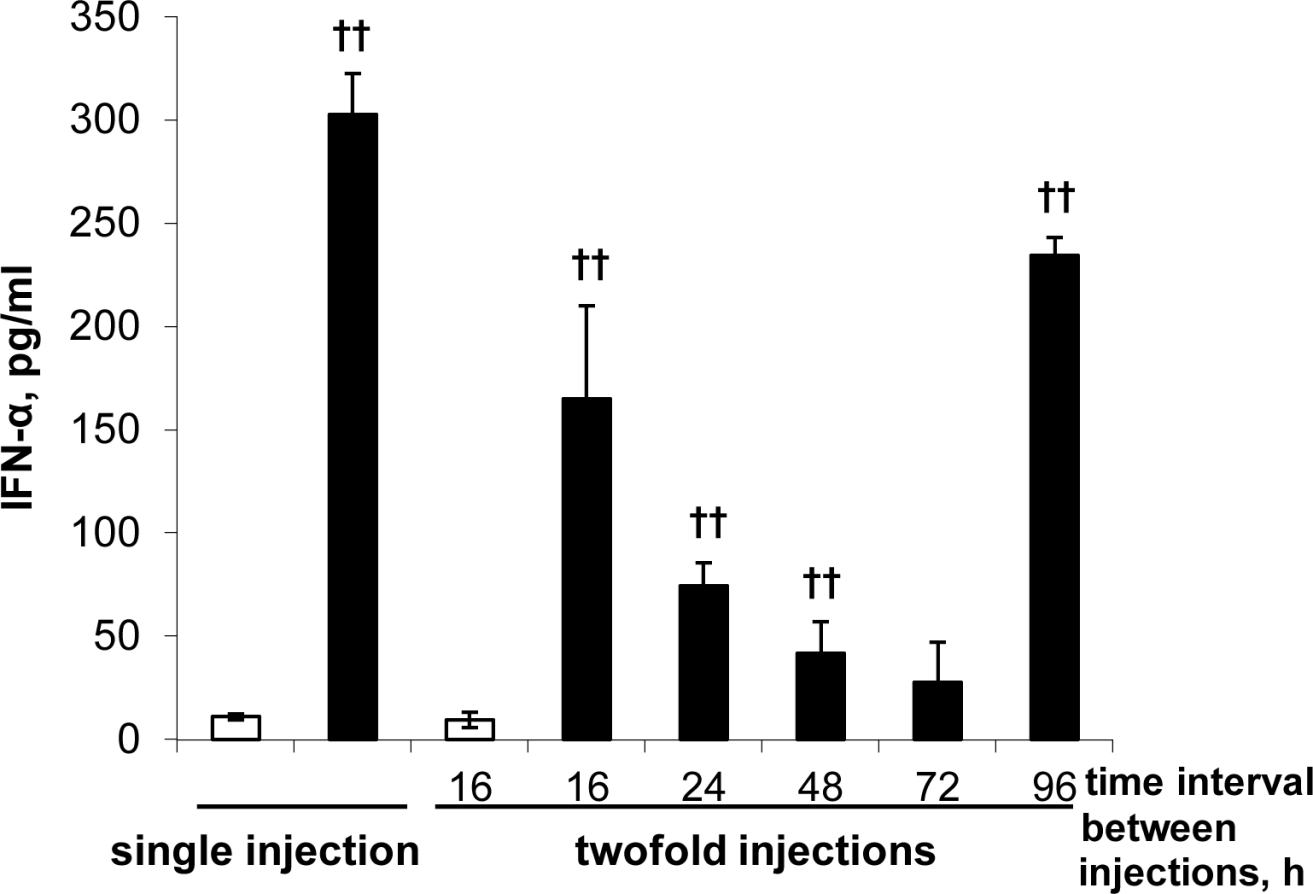
Refractory state for interferon induction follows a single injection of isRNA in mice. C57BL/6 mice were intravenously injected once or twice with 2X3-DOPE (white bars) or with isRNA/2X3-DOPE (black bars). The time intervals between the injections varied from 16 to 96 h. Serum IFN-α levels were measured by ELISA 6 h after the last injection. The data represent mean ± standard deviation (SD) calculated from measurements from at least three mice. Statistically significant differences between experimental groups and the 2X3-DOPE-treated group (Mock) are indicated by crosses (††, P<0.01); Mann-Whitney U test.

It has been previously shown that some short double-stranded RNA (e.g. siRNA) have immunostimulating effect which is mediated by the nucleic acid sensors (TLRs, RIG-I, etc.), and is accompanied by the production of type I IFNs [16, 27, 28] and a variety of other cytokines [29]. It has also been shown that tumor growth inhibition induced by TLR agonists critically depends on the production of type I IFNs [8]. Previously we have shown [23, 24] that isRNA under study strongly increased the level of IFNα, to a lesser extent the level of IL-6, and did not affect the level of IFNγ and TNFα in blood serum of C57Bl/6 and CBA/LacSto mice. Induction of IFNα was transient: The peak increase of the IFN-α level in murine serum was detected 6 h after i.v. administration of isRNA and 18 h later the IFN-α level was reduced to the initial one [23]. It is known that interferon synthesis is followed by a period of refractoriness during which restimulation with inducer (e.g. poly(I:C) and viral RNA) does not stimulate IFNα synthesis and secretion [30, 31]. To optimize the treatment regimen we determined the duration of the refractory state of IFNα production to restimulation by isRNA. Here the effect of repeated isRNA administrations on subsequent interferon-α production in C57Bl/6 murine blood serum was studied. Groups of C57Bl/6 mice were intravenously injected once or twice with isRNA/2X3-DOPE, or with 2X3-DOPE only (Mock). The time intervals between the two injections varied from 16 to 96 h. The IFN-α level was measured 6 h after the second injection. A single injection of isRNA induces ~30-fold increase in the IFN-α level relative to the level of Mock-injected mice (Fig 1). When the second isRNA/2X3-DOPE injection was made at a 16 h interval after the first one, the IFN-α level also increased substantially but to a lesser extent than after the first administration. When the time intervals between the injections were from 24 to 72 h the level of IFN-α was lower than in the group of mice after single injection of isRNA (Fig 1), possibly due to the formation of the interferon refractory state. The prolongation of the time interval between injections up to 96 hours resulted in reactivation of IFN-α synthesis and ~24-fold increase in the IFN-α level relative to the level of Mock-injected mice. It should be noted that repeated Mock injections do not affect the level of IFN-α. Based on the obtained data, we applied the scheme with 96 h time intervals between injections of isRNA/2X3-DOPE for the treatment of B16 melanoma-bearing mice.

### Antitumor effect of isRNA

**Fig 2:**
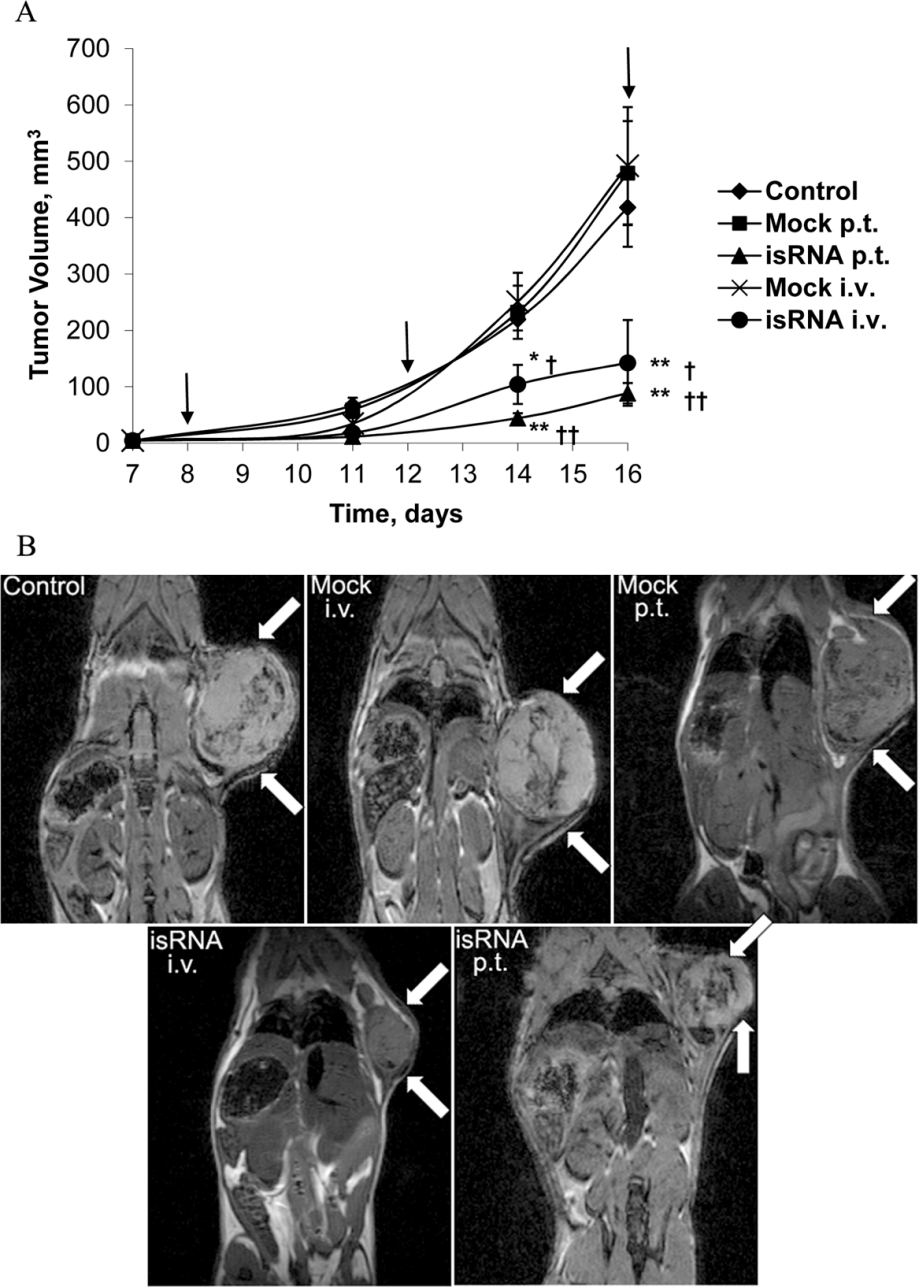
Treatment of melanoma B16-bearing mice with isRNA inhibits primary tumor growth. C57BL/6 mice with subcutaneous melanoma (1.5 × 105 cells per mouse) were treated with isRNA/2X3-DOPE complexes (p.t. or i.v. injections). (A) Dynamics of tumor growth. Black arrows indicate days of isRNA administration. The data represent mean ± SEM (n = 8–10). Statistically significant differences between experimental groups and the untreated group (control) are indicated by asterisks (**, P<0.01; *, P<0.05); and between experimental groups and the 2X3-DOPE-treated group by crosses (††, P<0.01; †, P<0.05); Mann-Whitney U test. (B) MR images of the representative mice from different groups on day 17 after tumor initiation. Frontal longitudinal sections with the maximum size of tumors from each group are shown. White arrows indicate tumor nodes.

We evaluated the antitumor activity of the isRNA/2X3-DOPE in mice with subcutaneous melanoma. B16 cells were injected subcutaneously into C57Bl/6 mice; starting on day 8 after tumor inoculation when the tumors reached 2–4 mm in diameter, i.v. or p.t. injections of isRNA/2X3-DOPE complexes, as well as Mock injections, were performed. The treatment was repeated on days 12 and 16. The data on the dynamics of primary tumor growth demonstrate (Fig 2A) that both i.v. and p.t. injections of isRNA/2X3-DOPE significantly inhibited tumor size (P<0.05, P<0.01, respectively) in comparison with control or Mock-treated mice. On day 16, the tumor volume in the control group reached 500 mm^3^, while in the isRNA-treated mice the tumor volume was 3 to 5 times smaller than in the Mock-treated animals and the control group. On day 17, serial MR image slices were taken of the animals (Fig 2B). The MRI data showed that the tumor size had decreased significantly in the mice that received p.t. or i.v. injections of isRNA/2X3-DOPE complexes in comparison with the Mock and control groups. The tumor size in the mice that received 2X3-DOPE according to the same dosing scheme did not differ from the tumor size in the untreated mice. Thus, isRNA/2X3-DOPE administered p.t. or i.v. efficiently inhibited the growth of subcutaneous melanoma. The experiment was terminated on day 18 because the tumor size in the control and Mock-treated groups had reached the ethical limit.

### Antimetastatic effect of isRNA

**Fig 3:**
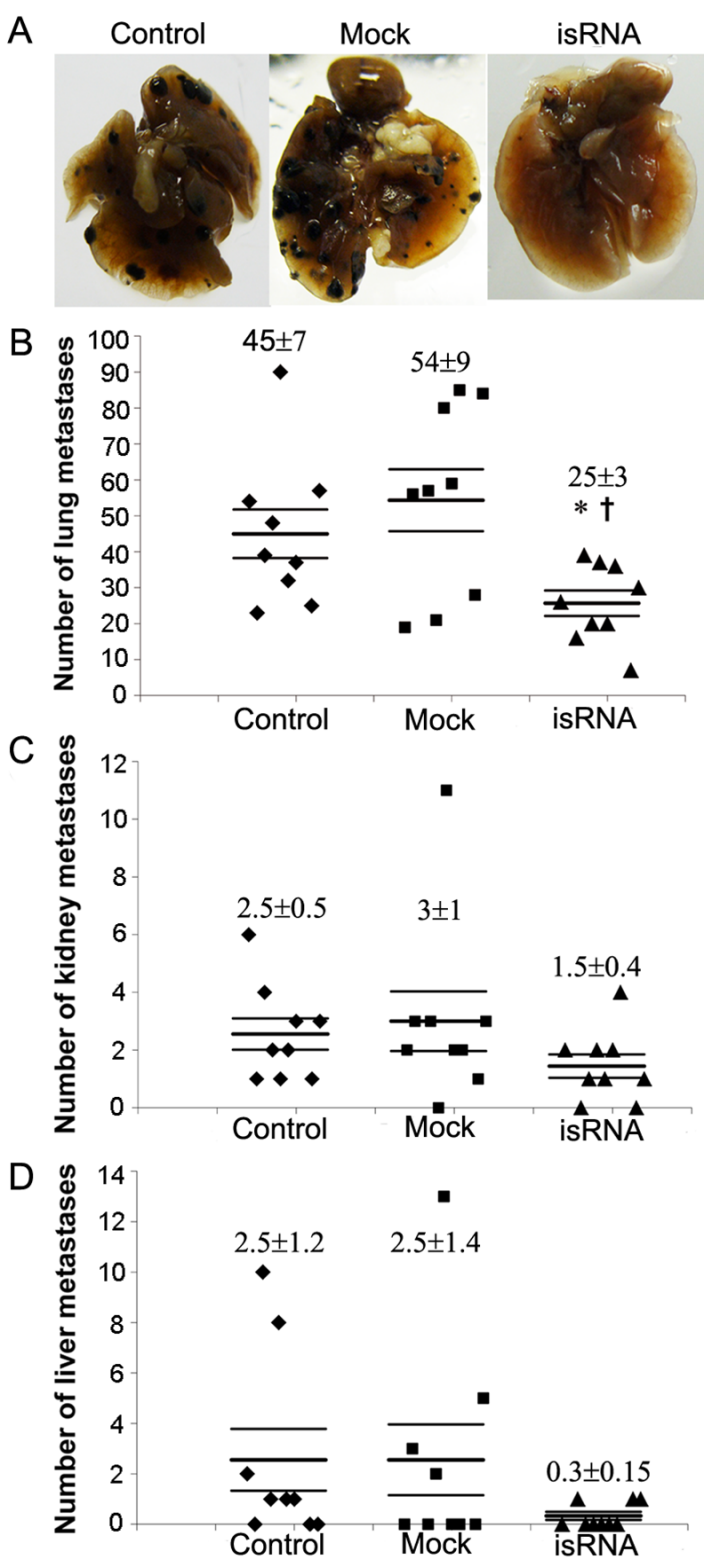
isRNA potently reduces the number of surface B16 melanoma metastases in different organs of mice. C57BL/6 mice were i.v. inoculated with melanoma B16 cells (2 × 105 cells per mouse) and treated with isRNA/2X3-DOPE complexes by i.v. administration on days 2, 6 and 10. (A) Images of lungs of representative mice from different groups on day 15 after tumor cell implantation. The numbers of macroscopically visible melanoma metastases in the lung (B), kidney (C) and liver (D), mean ± SEM, n = 9. Statistically significant differences between the experimental group and untreated group (control) are indicated by an asterisk (*, P<0.05); and between the experimental and Mock groups by a cross (†, P<0.05); Mann-Whitney U test.

The ability of isRNA/2X3-DOPE complexes to inhibit metastasis spreading was tested in a metastatic B16 melanoma model. The treatment was carried out on days 2, 6 and 10 after i.v. tumor cell inoculation, according to the previously selected time interval between the injections. Data displayed in Fig 3A and 3B show that systemic administration of isRNA/2X3-DOPE significantly reduces the number of surface metastases in lungs. The average number of metastases in the lungs of treated animals was 25±3, which was 2-fold lower than in the control (45±7) and Mock-treated (54±9) (P<0.05) groups. Surface metastases were also detected in the liver and kidneys of mice with metastatic B16 melanoma, but in lower amounts. Treatment with isRNA/2X3-DOPE reduces the number of kidney and liver metastases however, these differences were not statistically significant, due to the low number of metastases in those organs (the mean value for each group was from 0.3 to 3).

**Fig 4:**
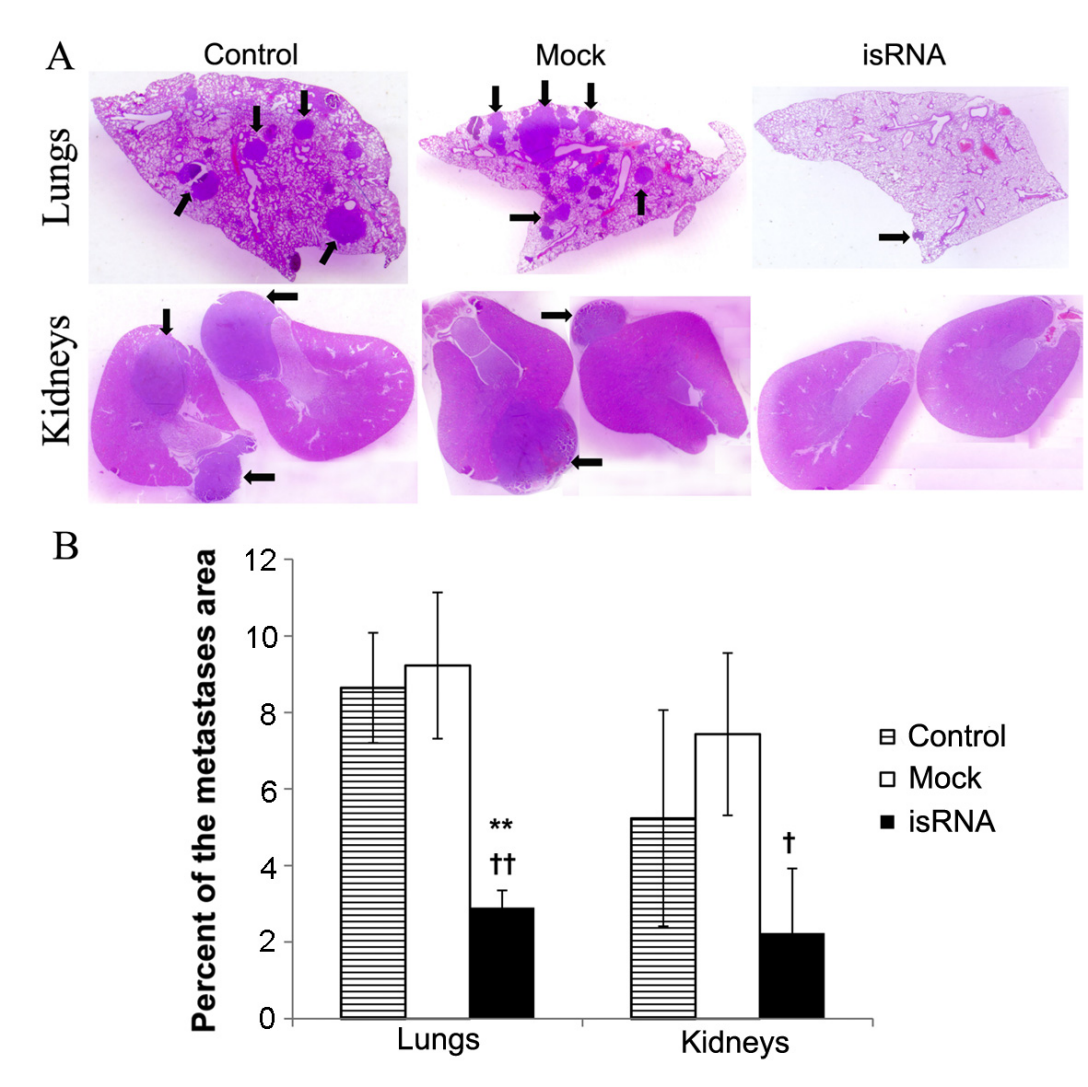
Treatment of melanoma B16-bearing mice with isRNA reduces the area of internal metastases in lungs and kidneys. (A) Images showing internal lung metastases (top row) and kidney metastases (lower row) of the representative mice from different groups on day 15 after intravenous B16 tumor cell implantation. Arrows show the metastases. Paraffin sections, hematoxylin and eosin stain. (B) The ratio of the total metastases area to the total area of the organ. The data represent mean ± SEM (n = 9). Statistically significant differences between experimental and control groups are indicated by asterisks (** P<0.01, * P<0.05); and differences between experimental and Mock groups are indicated by crosses (†† P<0.01, † P<0.05); Mann-Whitney U test.

The areas occupied by internal metastases in different visceral organs were measured to confirm the antimetastatic activity of isRNA/2X3-DOPE. Morphometric analysis of cross sections (Fig 4A and 4B) showed that isRNA/2X3-DOPE evidently reduces the average metastases area in lungs and kidneys: A 3-fold decrease in the internal metastases area in the lungs (P < 0.01) and kidneys (P < 0.05) was observed after isRNA treatment (Fig 4B). Internal metastases in the liver were not found. Overall, isRNA/2X3-DOPE significantly decreases the number of surface metastases in lungs and reduces the internal metastases area in kidneys and lungsof mice with metastatic B16 melanoma.

### isRNA induces remodeling of spleen

**Fig 5:**
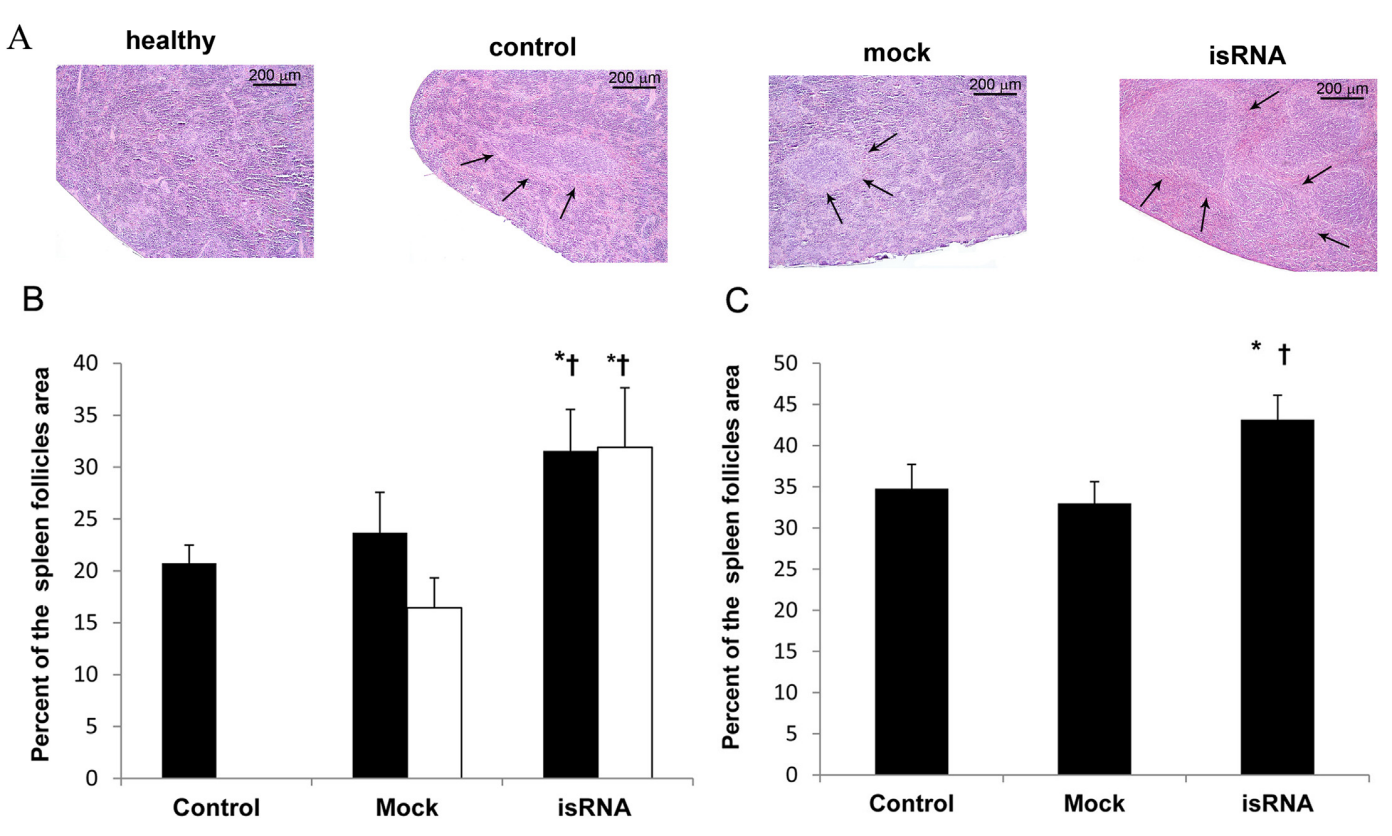
The treatment of melanoma B16-bearing mice with isRNA increases the white pulp area in the spleen. (A) Cross sections of the spleens of the representative mice with subcutaneous melanoma on day 18 after tumor initiation. Arrows show follicles on each image. Paraffin sections, hematoxylin and eosin stain. (B, C) The ratio of the total follicles area to the total area of the spleen for subcutaneous (B) and metastatic (C) melanoma (for details see Materials & Methods). I. v. administration–black bars, p.t. administration–white bars. The data represent mean ± SEM (n = 9). Statistically significant differences between experimental and control groups are indicated by asterisks (**, P<0.01; *, P<0.05); and differences between experimental and Mock groups are indicated by crosses (††, P<0.01; †, P<0.05); Mann-Whitney U test.

Morphological analysis of the spleen sections was performed to evaluate the immunostimulatory effect of isRNA/2X3-DOPE expressed in white pulp expansion in mice compared to the Mock and control groups of melanoma-bearing mice. It was shown that the character of the morphological changes is independent of tumor localization. Thus, using both subcutaneous and metastatic melanoma models (Fig 5A), a slight increase in the size and number of white pulp follicles with germinal centers was observed in the spleens of untreated mice in comparison with healthy mice. Additionally, a moderate venous hyperemia of blood vessels and red pulp of the spleen in the control group were observed. Both p.t. and i.v. administration of the isRNA/2X3-DOPE complexes significantly changed the splenic architecture, namely by increasing the size and number of splenic follicles, inducing their fusion with each other and forming large germinal centers in comparison with untreated and Mock-treated animals (Fig 5). The alterations in tissue architecture suggested immune system activation and enhanced antigen processing in the spleen [32]. It should be noted that no significant toxic effects on the spleen tissues were observed in the groups under study.

Morphometric analysis of spleen cross sections showed that isRNA treatment reliably increased (P < 0.05) the average white pulp area in mice with both metastatic and subcutaneous melanoma (Fig 5B and 5C). In the case of subcutaneous melanoma, the average white pulp area in the spleens of both i.v.- and p.t.-treated animals was increased to 32±4% in comparison with the control (20.5±4%) and Mock-treated (16.5±3% for p.t. and 23.5±4% for i.v. administration) groups. Similarly, in the metastatic melanoma model, the average white pulp area in the spleen of isRNA-treated animals was higher (43.2±3%) than that of the control (34.5±3%) and Mock (33±2.5%) groups (Fig 5B and 5C). The fact that isRNA treatment increased by 8–15% the white pulp area of the spleen of mice with both metastatic and subcutaneous melanoma confirms effective immunostimulation.

### The isRNA does not induce hepatotoxicity

**Fig 6:**
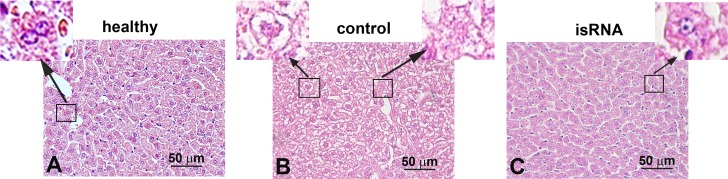
The effect of isRNA on the liver of mice with melanoma B16. Representative histological images of liver tissues of healthy animals (A), untreated animals with subcutaneous melanoma (B) and B16-bearing animals treated with p.t. injections of isRNA/2X3-DOPE complexes (C). Insets show binuclear hepatocytes (left), necrosis in the liver parenchyma (center) and hepatocytes with dystrophy (right). Hematoxylin and eosin stain, original magnification x400.

According to histopathological analysis of the liver tissue of untreated tumor-bearing mice, the tumor has a moderate toxic effect on the liver and increases the development of destructive and dyscirculatory changes (Fig 6). Disturbed circulation, dilatation and congestion in the central vein, hepatic sinus and interlobular veins, destruction of the hepatic lobule structure, protein degeneration of hepatocytes, and frequent monocellular and focal necrosis were observed in the livers of tumor-bearing mice. The results revealed (Table 1) that by day 15–18 of tumor progression the normal liver tissue occupied only ~52%, whereas tissue with degenerative and necrotic changes constitutes 20.6 and 21.5% in the liver of mice with subcutaneous melanoma, and 14.4 and 30.4% in the liver of mice with metastatic melanoma, respectively (Table 1). In the group of Mock-treated mice the percentage of dyscirculatory and destructive changes in the liver parenchyma varies depending on the route of administration: Does not change after p.t. administration and slightly increases after i.v. administration in comparison with the control group (Table 1).

**Table 1:** The effect of isRNA treatment on the liver of B16 melanoma-bearing mice.

	Healthy	C57Bl mice with subcutaneously implanted B16					C57Bl mice with intravenously implanted B16		
	C57Bl	Control ^a^ (untreated)	Mock ^b^p.t. ^d^	isRNA ^c^p.t. ^d^	Mock ^b^i.v. ^e^	isRNA ^c^i.v. ^e^	Control ^a^ (untreated)	Mock ^b^i.v. ^e^	isRNA ^c^i.v. ^e^
**Normal liver parenchyma, Vv** ^f^**, %**	84.6±0.6	52.6±2.3	53.5±3.4	64.6±2.9*†	44.5±1.7*	54.5±4.3#	51.8±3	45.6±4.9	51±2.6
**Dystrophy, Vv** ^f^**, %**	4.7±0.5	20.6±1.2	18.3±1.7	9.9±1.6*†	17.5±0.4*	17±3.6	14.4±2.6	24.1±3.5*	19.2±1.9*
**Necrosis, Vv** ^f^**, %**	6.2±0.7	21.5±0.8	23.3±1.8	19.2±1.9	31.3±1.6*	20.7±1.8#	30.4±2	25.8±2.2*	24.3±1.5*
**Total destructive changes, Vv** ^f^**, %**	10.9±1.2	42.1±1.8	41.6±3.5	29.2±4*†	48.7±1.2*	37.7±4.9#	44.7±3	49.9±5.1*	43.5±2.9
**Binuclear hepatocytes, Nv** ^g^	2±0.4	3.2±0.3°	2.4±0.3*	2±0.3*	2.4±0.7	1.9±0.7*	1.6±0.5	1.8±0.8	0.9±0.4 °

^a^ Control—group of mice received saline buffer injections.

^b^ Mock—group of mice received 2X3-DOPE in OptiMEM.

^c^ isRNA—group of mice received isRNA/2X3-DOPE complexes in OptiMEM.

^d^ Peritumoral (p.t.) injections of preparations used for tumor treatment.

^e^ Intravenous (i.v.) injections of preparations used for tumor treatment.

^f^ The volume density (Vv) representing the volume fraction of tissue occupied by this compartment.

^g^ The numerical density (Nv) indicating the number of particles in the unit tissue volume.

* statistically significant difference relative to C57Bl bearing B16, p ≤ 0.05

† statistically significant difference relative to the peritumoraly injected Mock group, p ≤ 0.05; # statistically significant difference relative to the intravenously injected Mock group, p ≤ 0.05

° statistically significant difference of binuclear hepatocytes relative to healthy C57Bl, p ≤ 0.05.

Treatment of subcutaneous melanoma with isRNA/2X3-DOPE (Fig 6, Table 1) leads to a decrease in the volume density of dystrophy and necrosis of the liver parenchyma; the obvious positive effect was observed after both p.t. and i.v. administration of the preparation. When isRNA/2X3-DOPE was injected peritumorally the normal parenchyma area reliably increased to 64.6% (P ≤ 0.05) in comparison with 52.6 and 53.5% in the liver of untreated and Mock-treated mice, respectively (Table 1). isRNA/2X3-DOPE administered intravenously also reliably increased the normal liver parenchyma area to 54.5% in comparison with Mock-treated mice (44.5%), but this parameter did not differ significantly from the untreated groups of mice (52.6%) (Table 1). The liver regenerative activity was evaluated by calculation of the numerical density of binuclear hepatocytes (Table 1). The growth and development of primary tumors reliably increased (P ≤ 0.05) the numerical density of binuclear hepatocytes in the liver of untreated B16-bearing mice to 3.2 compared with 2 in the livers of healthy animals (Table 1). Peritumoral or intravenous administration of isRNA/2X3-DOPE or 2X3-DOPE alone in tumor-bearing mice reduced the regenerative activity of the liver to the level of healthy animals.

The treatment of metastatic melanoma with isRNA/2X3-DOPE did not change either the normal parenchyma area (about 51%) or the total destructive changes (43.5–44.7%) in the liver tissue, but reduced the severity of destructive changes. Thus, isRNA/2X3-DOPE increases the proportion of less severe and reversible dystrophy of hepatocytes (19.2% for isRNA-treated versus 14.4% for control groups) and decreases the proportion of necrosis (24.3% for isRNA-treated versus 30.4% for control groups) (Table 1). Again, the injections of 2X3-DOPE alone slightly increased the hepatotoxicity in tumor-bearing mice, whereas in healthy mice no signs of hepatotoxicity induced by 2X3-DOPE during acute or chronic tests were found (data not presented). The presence of isRNA in the complex with 2X3-DOPE eliminates the negative impact (if any) of the vehicle (Table 1). Calculation of the numerical density of binuclear hepatocytes showed that the development of metastases in mice causes an insignificant reduction in the numerical density of binuclear hepatocytes in untreated mice and more pronounced 2-fold reduction in the livers of isRNA-treated mice compared with healthy animals (Table 1). Summing up the above data it can be concluded that both i.v. and p.t injections of isRNA/2X3-DOPE reduce the toxic effects on the liver caused by tumor progression.

## Discussion

Immunotherapy is an attractive approach in cancer therapy that has been intensively studied in recent years in the context of cancer treatment or prevention [33]. Various types of immunostimulatory nucleic acids are tested as potential drugs for the treatment of neoplastic diseases, including such nucleic acids as long dsRNAs [34, 35], CpG containing oligonucleotides [36–41], siRNAs with immunostimulatory motives [15, 16, 28, 42], oligoribonucleotides with triphosphate at the 5’ end [11, 43, 44] and different chimeric molecules containing immunostimulatory domain [45]. In their study, Poeck et al. [11] showed that innate immune activation relied on the recruitment of RIG-I by the addition of triphosphate to the 5’ ends of oncogene-specific siRNAs. Using a slightly different approach, two groups [38, 39] have demonstrated that conjugation of anti-STAT3 siRNA with CpG ODN, a TLR9 agonist, resulted in potent antitumor effects. Antitumor activity occurs via the induction of type I IFNs, the activation of tumor-resident macrophages and the restoration of normal immune function. In a recent study, it was shown by Khairuddin et al. [28] that activation of the innate immune response by siRNAs has significant antitumor effects against HPV-driven tumors, even in the absence of a specific gene target. These findings imply the potential prophylactic and therapeutic use of immunostimulatory RNAs as adjuvants.

The present study used a specific 19-bp isRNA duplex with 3’ 3-nt overhangs and examined its immunostimulatory, antitumor and antimetastatic activities in a melanoma B16/C57Bl/6 mice tumor model. The isRNA under study is one nucleotide longer than canonical small interfering RNAs and has no significant homology with any mRNAs from mice and humans; therefore it does not act via the RNAi mechanism, but induces activation of the innate immune system. Previously, we applied this isRNA for the treatment of hepatocellular carcinoma G29 and showed that isRNA reduced the metastases area in the different organs of G29-bearing mice and slightly inhibited primary tumor growth [24].

B16 melanoma is a highly aggressive and fast-growing tumor so we were faced with two objectives: (1) to achieve the effective delivery of isRNA into immune and maybe into tumor cells *in vivo*, and (2) to select the effective dosing scheme.

We used recently developed 2X3-DOPE for the *in vivo* delivery of isRNA, this lipid provided an effective delivery of siRNA into tumor cells and the presence of serum in the medium did not affect its transfection activity [25]. Previously, we have shown that 2X3-DOPE provided delivery of fluorescein-labeled ODN, plasmid DNA and siRNA into HEK293, BHK cells in vitro [25] and into DCs ex vivo [46] with an efficiency significantly higher than that of Lipofectamine 2000, known as the gold standard for the delivery of nucleic acids. Moreover, isRNA/2X3-DOPE complexes administered intravenously induced the production of IFN-α level in murine blood serum more effectively than isRNA/Lipofectamine 2000 complexes (data are not shown). Here, we show that treatment of mice with subcutaneous and metastatic melanoma B16 by isRNA/2X3-DOPE did not cause hepatotoxicity in mice. On the contrary, isRNA/2X3-DOPE reduces the area and severity of destructive changes in the liver caused by tumor development, and does not reduce the regenerative activity of the liver below than in healthy mice demonstrating the safety of its application.

Selection of the dosing regimen is particularly important for therapy with interferon inducers in connection with the formation of an interferon refractory state. As mentioned above, after stimulation by an interferon inducer, interferon synthesis is followed by a period of refractoriness during which restimulation with the inducer fails to stimulate an interferon response [30, 31]. Cross-refractoriness to different inducers of IFN-α is recognized, while signaling by other types of interferons (β and λ) is not affected during repeated stimulation of the IFN signal transduction pathway [47]. In addition to the refractoriness to interferon synthesis, there are mechanisms responsible for the refractoriness to interferon signaling in the cell [48] and elimination of interferon [49]. Unfortunately, there is no clear understanding of all mechanisms of formation of the interferon resistance state and not all molecules responsible for its formation have been identified yet [49]. However, according to different data the duration of the interferon refractory state varied from 72 h to 13 days [30].

Different treatment schemes for the administration of interferon inducers are described in the literature: The most frequent intervals between injections are 72 h [8, 11, 37] and 96 h [28, 36, 37]. We determined experimentally the duration of the refractory state of IFNα synthesis to reinduction by isRNA/2X3-DOPE to optimize the treatment regimen. The results revealed (Fig 1) that the reactivation of IFN-α synthesis occurs at a time interval of 96 h between injections, while the use of shorter time intervals did not result in activation of IFNα production. A similar treatment regimen was used by Najar et al. [36], who treated malignant melanoma in mice by CpG ODN in combination with dacarbazine and treatment cycles were repeated every 96 hours. In two other studies authors used CpG ODN [37] or immunostimulatory siRNA [28] for the tumor treatment, and time intervals between injections were different: The first interval was 72 hours and the subsequent intervals were 96 hours. Previously we applied the scheme with 72 h time intervals between injections on an HCC G-29 [24] and melanoma B16 (data are not shown) mice models, isRNA under study provided good inhibition of metastases, while the effect on the primary tumor node was insignificant. Taking into account our new results, we can conclude that in the previous scheme each second injection was useless. In the present study, we show that three doses of isRNA/2X3-DOPE with 96 h time intervals between injections significantly inhibited B16 primary tumor growth (up to a 5-fold reduction of the tumor volume) and the metastases spreading (2–4-fold reduction in the number and area of metastases) in comparison with the control groups on the 14th–16th day of tumor progression.

The literature data on the application of the immunostimulatory nucleic acids (e.g. TLR agonists) in the treatment of melanoma are split among therapies in which the agonists are delivered systemically, namely intravenously [11, 28] and intraperitoneally [9, 37, 40], or locally to the tumor environment [36, 38, 41, 50]. In the present study we used intravenous and peritumoral injections of isRNA/2X3-DOPE for the treatment of subcutaneous melanoma in mice and both of them were equally effective in terms of tumor growth inhibition. Liver injury in tumor-bearing subjects, including subcutaneous B16-bearing mice, could occur due to both metastatic infiltration of the liver, and accumulation in the liver of T cells and myeloid derived suppressor cells (MDSC) [51]. Our results showed that in addition to antitumor activity, isRNA/2X3-DOPE has a positive effect on the liver of melanoma-bearing animals: Reduces the volume density of destructive changes in the liver parenchyma and maintains the regenerative activity of the liver. Interestingly, the reduction of destructive changes in the liver parenchyma was more pronounced after p.t. than after i.v. administration of isRNA/2X3-DOPE. These data are in agreement with the results obtained by two groups of authors who used other types of immunostimulating nucleic acids [36, 52] and highlighted the therapeutic potential of TLR agonists to function better on a local level, rather than on a systemic one, for the treatment of subcutaneous melanoma. For example, Amos and coworkers showed that the delivery of the TLR agonists poly(I:C) and CpG ODN (25 μg of each per mice) via systemic routes exerted similar antitumor effects to those achieved via local administration, but was associated with adverse effects [34]. In our study, isRNA showes no adverse side effects and even displays a hepatoprotective effect. Perhaps it is due to the fact that isRNA after i.v. administration mainly induces the synthesis of IFNα, to a lesser extent the synthesis of IL-6 [23] and does not induce TNF-α (data are not shown) unlike the other inductors based on long dsRNA [34, 35] and CpG oligonucleotides [53]. Moreover, there are evidences in literature that tumor growth inhibition induced by dsRNA or poly(I:C) critically depends on the production of type I IFNs and is accompanied by elevation of tumor-specific CD8(+) and CD4(+) T cell number [8, 54]. Our data (see S1 Table, S1 Fig) also show increased number of T cells in the tumor under the action of isRNA.

The spleen is the most important organ of the secondary lymphatic system and includes white pulp (lymphocytes) and red pulp (sinuses and blood vessels). The white pulp is involved in the induction of immune responses and antibody production; white pulp expansion is important for secondary immune responses and adaptive immunity [55]. Histological analysis of spleen cross sections of B16-bearing mice showed profound splenic remodeling such as hyperplasia of the lymphoid white pulp and the formation of large germinal centers after isRNA/2X3-DOPE administration, suggesting immune activation and enhanced antigen processing and B lymphocyte proliferation in the spleen, confirming the immunostimulatory action of isRNA.

In summary, in this work we demonstrate that isRNA/2X3-DOPE displays efficient immunostimulatory, antitumor and antimetastatic properties in mice with melanoma B16. It has no apparent toxic effects on the organs of mice and even displays a hepatoprotective effect. Thus, the studied isRNA/2X3-DOPE can be considered a potential drug or adjuvant for the therapy of melanoma B16 and other immunosuppressive diseases.

## Supporting Information

S1 Fig Infiltration of melanoma B16 primary tumor node by CD8 lymphocytes (immunohistochemical staining). Representative images of tumor sections obtained from animals with s.c. implanted melanoma B16 without treatment (A), B16-bearing animals treated with p.t. injections of 2X3-DOPE (B) and B16-bearing animals treated with p.t. injections of isRNA/2X3-DOPE complexes (C). Arrows show CD8 lymphocytes in the area between normal and necrotic tumor tissue. Immunohistochemical staining of paraffin sections by CD8. Magnification ×400.
(DOCX)

S1 Table Infiltration of melanoma B16 primary tumor node by CD4 and CD8 lymphocytes (Vv, %). (DOCX)
