# Supplementary material for: Antitumor and Antimetastatic Effect of Small Immunostimulatory RNA against B16 Melanoma in Mice
Source: PLoS One. 2016 Mar 16;11(3):e0150751. doi: 10.1371/journal.pone.0150751 (PMC4794162; doi:10.1371/journal.pone.0150751)
Supplement: S1 Fig — Representative images of tumor sections obtained from animals with s.c. implanted melanoma B16 without treatment (A), B16-bearing animals treated with p.t. injections of 2X3-DOPE (B) and B16-bearing animals treated with p.t. injections of isRNA/2X3-DOPE complexes (C). Arrows show CD8 lymphocytes in the area between normal and necrotic tumor tissue. Immunohistochemical staining of paraffin sections by CD8. Magnification ×400. (DOCX) [file pone.0150751.s001.docx]

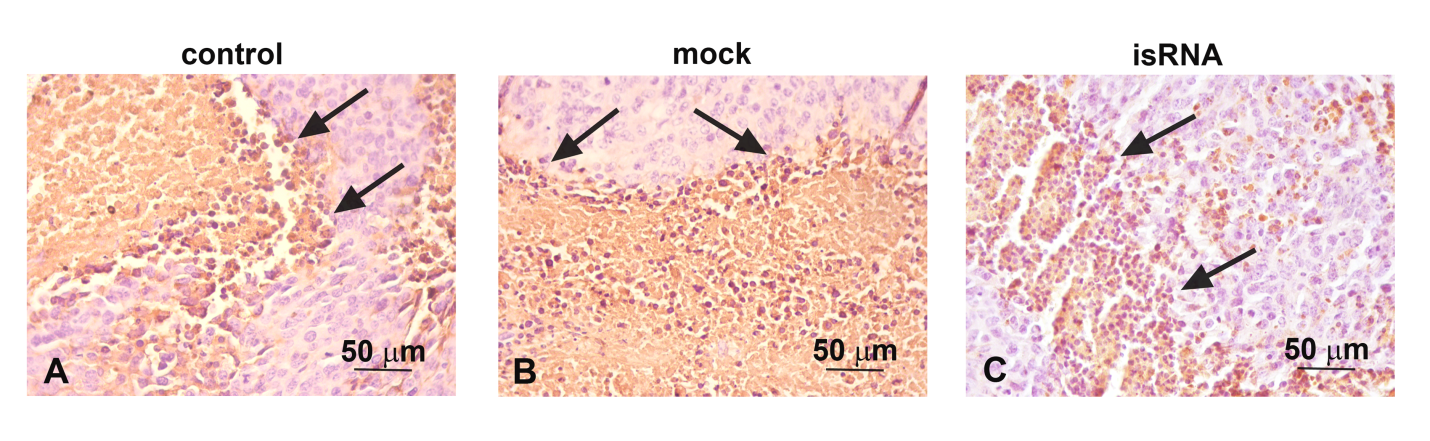


**S1 Fig. Infiltration of melanoma B16 primary tumor node by CD8 lymphocytes** (immunohistochemical staining). Representative images of tumor sections obtained from animals with s.c. implanted melanoma B16 without treatment (A), B16-bearing animals treated with p.t. injections of 2X3-DOPE (B) and B16-bearing animals treated with p.t. injections of isRNA/2X3-DOPE complexes (C). Arrows show CD8 lymphocytes in the area between normal and necrotic tumor tissue. Immunohistochemical staining of paraffin sections by CD8. Magnification *×*400.
