# Supplementary material for: Antitumor and Antimetastatic Effect of Small Immunostimulatory RNA against B16 Melanoma in Mice
Source: PLoS One. 2016 Mar 16;11(3):e0150751. doi: 10.1371/journal.pone.0150751 (PMC4794162; doi:10.1371/journal.pone.0150751)
Supplement: S1 Table — (DOCX) [file pone.0150751.s002.docx]

**S1 Table.** **Infiltration of melanoma B16 primary tumor node by CD4 and CD8 lymphocytes (Vv, %).**

|  | CD4 | | | CD8 | | |
| --- | --- | --- | --- | --- | --- | --- |
|  | Mean* | p value | | Mean* | p value | |
|  |  | vs control | vs mock |  | vs control | vs mock |
| control | 4.87 |  |  | 21 |  |  |
| mock p.t. | 4.07 |  |  | 39.7 | 0.000055 |  |
| isRNA p.t. | 5.0 |  |  | 27.76 |  | 0.007 |
| mock i.v. | 4.0 |  |  | 31.53 | 0.016 |  |
| isRNA i.v. | 7.93 | 0.000374 | 0.000058 | 39.63 | 0.000003 | 0.047 |

*Data of histological staining of tumor samples with anti-CD8 and anti-CD4 mAb were statistically processed using one-way ANOVA. Post-hoc testing was completed using Fisher’s least significant differences (LSD). P <0.05 was considered to be statistically significant. Statistical package STATISTICA version 10.0 has been used for analysis.
